# Correlation of clinical signs and symptoms of Behçet’s disease with mean platelet volume (MPV) and red cell distribution width (RDW)

**DOI:** 10.1186/s13023-020-01588-1

**Published:** 2020-10-21

**Authors:** Maryam Masoumi, Soraya Shadmanfar, Fereydoun Davatchi, Farhad Shahram, Massoomeh Akhlagi, Tahereh Faezi, Hoda Kavosi, Soroush Moradi, Javad Balasi

**Affiliations:** 1 Clinical Research and Development Center, Shahid Beheshti Hospital, Qom University of Medical Sciences, Beheshti Blvd, 3719964797 Qom, Qom Iran; 2 Tehran University of Medical Sciences, Tehran, Iran; 3 Rheumatology Research Center, Tehran University of Medical Sciences, Tehran, Iran; 4 Student Research Committee, School of Medicine, Iran University of Medical Sciences, Tehran, Iran

**Keywords:** Behçet’s disease, Mean platelet volume, Red blood cell distribution width, Uveitis, Ocular involvement

## Abstract

**Background:**

A strong correlation was previously found between mean platelet volume (MPV), red blood cell distribution width (RDW), and the severity of signs and symptoms in patients suffering from inflammatory and autoimmune diseases. The current study evaluated these correlations in patients with Behçet’s disease (BD) as well the relationship between MPV and RDW and disease activity score on the Iranian Behçet’s Disease Dynamic Activity Measurement (IBDDAM).

**Methods:**

This cross-sectional study included 319 patients with BD for whom demographic and epidemiological data, IBDDAM scores, and duration of illness was recorded. Blood samples were then obtained and the relationships between their disease status and manifestations and their laboratory parameters were evaluated with statistical models to find possible correlations.

**Results:**

Our analysis showed a significantly higher RDW in patients with BD who had ocular manifestations (*p* < 0.001) and oral aphthae (*p* = 0.004). Patients with active BD had higher RDW (*p* < 0.001) and MPV (*p* < 0.001) in comparison to those with currently inactive BD. Similarly, patients who had any type of ocular manifestation had higher RDW (*p* < 0.001) and MPV (*p* < 0.001). Regression analyses identified a statistically significant model for the effect of RDW and MPV in predicting active BD status (*p* < 0.001), as well as its significant relationship with active ocular manifestations (*p* < 0.001).

**Conclusion:**

BD was found to be associated with an increase in MPV and RDW, particularly during active phases. RDW and MPV were also found to have predictive value for screening to detect BD activity and its ocular complications.

## Background

Behçet’s disease (BD) is a multisystemic, chronic, relapsing inflammatory disorder characterized by recurrent ulcerations and other clinical manifestations [1]. The main clinical manifestations include involvement of the mucocutaneous, urogenital, locomotor, ocular, neurological, gastrointestinal, respiratory, and vascular systems [2, 3]. Among these, ocular involvement is considered one of the most prevalent symptoms of BD [4, 5]. Disease activity in BD is clinically assessed with several classification scales, including the Iranian Behçet’s Disease Dynamic Activity Measurement (IBDDAM), which evaluates disease activity according to clinical findings during the preceding 12 months [6]. However, there is still a demand for novel laboratory markers, not only to determine disease activity in patients with BD, but also to find potentially informative correlations with different signs and symptoms that can help to predict the disease course and prevent its exacerbation [7, 8]. As a result, biomarkers in blood samples from patients are currently receiving much attention in the search for possible correlations with diseases activity and its manifestations [9, 10].

In terms of its pathological manifestations BD represents a type of vasculitis; thanks to its specific clinical signs and symptoms, it is generally more easily diagnosed than other types of vasculitis since BD affects all vessels regardless of their size [11]. Although patients with BD may suffer from thrombosis-associated vascular involvement, the exact mechanism of thrombus formation remains unclear. A further consideration is that endothelial injury might not be solely responsible for the development of thrombosis, since several patients who have presented with thrombosis lacked endothelial injury [12]. Some studies suggested that platelet function correlates with the development of thromboses in patients with BD [9, 10]. With an accurate determination of the relationship between platelet activity and morphological features such as volume, mean platelet volume (MPV) might be able to serve as a diagnostic marker to further study the role of platelets in thrombosis formation [12, 13].

Mean platelet volume (MPV) is an important indicator of platelet activation. It increases with an increase in thrombocyte production. Important alterations in volume parameters of thrombocytes may have prophylactic and diagnostic importance in thrombotic events and prothrombotic tendency. In addition, increments in the platelet volume causes ischemia and infarction [14].

Red blood cell distribution width (RDW) is another parameter proposed to be associated with inflammatory processes. However, its use is mostly limited to the diagnosis of anemia because it is a numerical measurement of red blood cell (RBC) heterogeneity in volume in complete blood counts [15, 16].

Several earlier studies have shown a strong correlation of MPV and RDW with the severity of patients’ signs and symptoms, particularly in individuals suffering from inflammatory and autoimmune diseases including BD [17, 18]. In addition, a few studies have evaluated these markers in patients with ocular manifestations of BD although none of them has investigated the relationship between MPV and RDW with disease activity based on activity measurement tools. Therefore, the present study was designed to compare MPV and RDW in patients with ocular BD and those with other manifestations, as well as the correlation of these measures with disease activity score according to the IBDDAM.

## Methods

Patients diagnosed with BD were enrolled in this cross-sectional study in consecutive order, regardless of the current activity state of their disease. Only drug-naive patients had been recruited in our study. Patients suffering from other autoimmune or inflammatory conditions, including but not limited to malignancies and endocrine diseases, were excluded from this study.

BD was diagnosed according to the ICBD criteria.

The Ethics Committee of Tehran University of Medical Sciences approved the study protocol, and all patients provided informed consent before participation. The IBDDAM questionnaire outcomes were used only for investigational purposes and had no impact on clinical decision making or treatment decisions.

The patients’ demographics and clinical information including age, gender, duration of the disease, medications used (e.g. colchicine, prednisolone, cytotoxic drugs, and disease-modifying anti-rheumatic drugs [DMARDs]) were recorded, along with IBDDAM scores [21]. The patients’ IBDDAM scores were obtained in two follow-up sessions, and the overall score was calculated by adding the scores from the two sessions and dividing the sum by the interval between the two sessions (in months). The IBDDAM score evaluates 11 clinical manifestations, including oral aphthae (one point per five ulcers), genital ulceration (one point per ulcer), pseudofolliculitis (one point per ten lesions), erythema nodosum (one point per five lesions), arthritis (arthralgia one point, monoarthritis two points, polyarthritis three points), venous involvement (thrombophlebitis one point, large vessel thrombosis two points), intestinal manifestations (three points for mild manifestations, six points for moderate to severe manifestations), central nervous system (CNS) manifestations (one point for mild headaches, three points for mild CNS involvement, six points for moderate to severe manifestation), epididymitis (two points), and pathergy (one point). Then blood samples were obtained to record complete blood count (CBC) components. MPV and DW were also measured by same laboratories.

### Statistical analysis

Continuous variables were reported as the mean ± standard deviation (SD), and percentage and frequency values were used to report categorical data. Continuous and parametric data were compared between groups with an independent *t*-test, whereas categorical data were analyzed with the chi-squared test. A point-biserial correlation was run between CBC parameters of MPV and RDW and the use of DMARDs. Preliminary analyses showed no outliers, as assessed by boxplot, and engagement score was normally distributed, as assessed by Shapiro–Wilk’s test (*p* > 0.05). In addition, variances were homogeneous, as assessed by Levene’s test for equality of variances.

Multiple regressions were run to predict the IBDDAM score from MPV and RDW. Linearity was found in partial regression plots and a plot of studentized residuals against the predicted values. The independence of residuals was verified by a Durbin–Watson statistic of 1.873, and homoscedasticity was confirmed by visual inspection of a plot of studentized residuals versus unstandardized predicted values. No evidence of multicollinearity was found, given that the tolerance values were higher than 0.1. There were no studentized deleted residuals greater than ± 3 standard deviations, no leverage values greater than 0.2, and values for Cook’s distance were greater than 1. The assumption of normality was met, as assessed by a Q–Q Plot.

Binomial logistic regression was used to ascertain the effects of MPV and RDW on the likelihood that participants had active BD and active ocular manifestation of the disease. Linearity of continuous variables for the logit of the dependent variable was assessed with the Box–Tidwell procedure. The sensitivity, specificity, area under the curve (AUC), and optimal cut-off values were determined with receiver operating characteristic (ROC) curves. All analyses were done with SPSS Statistics for Windows version 25.0 (IBM Corp., Armonk, NY, USA). A *p* value less than 0.05 was considered statistically significant.

## Results

### Patients

Three hundred twenty patients with BD between 10 and 81 years old (mean age 43.5 ± 12 years) were enrolled. Of these, 142 patients (44.5%) were female and 177 (55.5%) were male. Their medical histories indicated that 157 patients used colchicine, 33 received prednisolone, and 130 underwent DMARD therapy with azathioprine (AZA) in 123 patients, methotrexate (MTX) in 97, and cyclosporine (CsA) in 16. Complete blood count showed average values of 88.01 ± 7.20 for MCV and 13.96 ± 1.35 for RDW.

### MPV and RDW in different BD manifestations

The prevalence of different clinical manifestations of BD is summarized in Table 1. An independent-sample *t*-test was run to determine whether differences in RDW or MPV corresponded with the appearance of specific BD signs and symptoms. Analyses showed that RDW was significantly higher only in patients with ocular manifestations (*p* < 0.001) and oral aphthae (*p* = 0.004). This, however, was not the case for MPV, and no correlation was found between MPV and any disease signs or symptoms.

**Table 1:** Mean platelet volume (MPV) and red blood cell distribution width (RDW) values according to different clinical manifestations of Behçet’s disease

BD manifestations		Frequency (%)	MPV (mean ± SD)	*p*	RDW (mean ± SD)	*p*
Oral aphthae	No	4 (1.3%)	8.9 ± 1.56	0.94	13.05 ± 0.31	0.25
	Yes	315 (98.7%)	10.45 ± 2.26		13.97 ± 1.36	
Genital aphthae	No	137 (42.9%)	10.42 ± 2.17	0.17	13.86 ± 1.38	0.04
	Yes	182 (57.1%)	10.43 ± 2.33		14.04 ± 1.34	
Ocular	No	68 (21.3%)	10.35 ± 1.98	0.74	13.48 ± 1.13	< 0.001
	Yes	251 (78.7%)	10.45 ± 2.33		14.09 ± 1.39	
Dermal	No	236 (74%)	10.38 ± 2.28	0.56	14.02 ± 1.37	0.18
	Yes	83 (26%)	10.55 ± 2.2		13.79 ± 1.32	
Pathergy test	No	200 (62.7%)	10.41 ± 2.27	0.84	14.02 ± 1.37	0.33
	Yes	119 (37.3%)	10.46 ± 2.24		13.87 ± 1.35	
Vascular	No	294 (92.2%)	10.41 ± 2.24	0.61	13.99 ± 1.37	0.19
	Yes	25 (7.8%)	10.65 ± 2.5		13.62 ± 1.14	
Gastrointestinal	No	305 (95.6%)	10.43 ± 2.27	0.94	13.97 ± 1.37	0.6
	Yes	14 (4.4%)	10.39 ± 2.05		13.78 ± 1.04	
CNS	No	315 (98.7%)	10.42 ± 2.27	0.1	13.96 ± 1.36	0.62
	Yes	4 (1.3%)	10 ± 0.3		13.57 ± 0.25	

BD, Behcet’s disease; CNS, central nervous system; MPV, mean platelet volume; RDW, red blood cell distribution width; SD, standard deviation

Laboratory test results were compared in patients taking different medications. In patients who were taking DMARDs, MPV was significantly higher than in patients who were not using this medication (10.78 ± 2.49 vs. 10.17 ± 2.05, *p* = 0.02). There was no statistically significant difference in RDW values between patients taking and not taking DMARDs (14.11 ± 1.39 vs. 13.85 ± 1.32, *p* = 0.09).

### Active BD manifestations

**Fig. 1:**
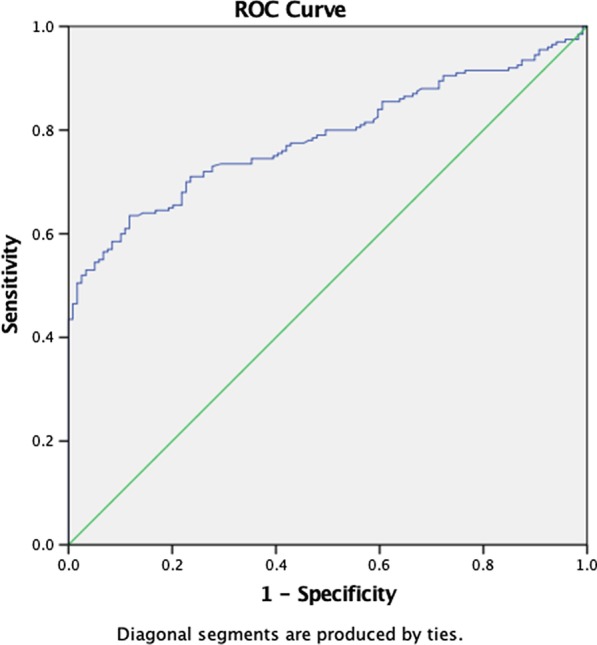
Receiver operating curve of red cell distribution width (RDW) and mean platelet volume (MPV) in patients diagnosed with active Behçet’s disease

**Fig. 2:**
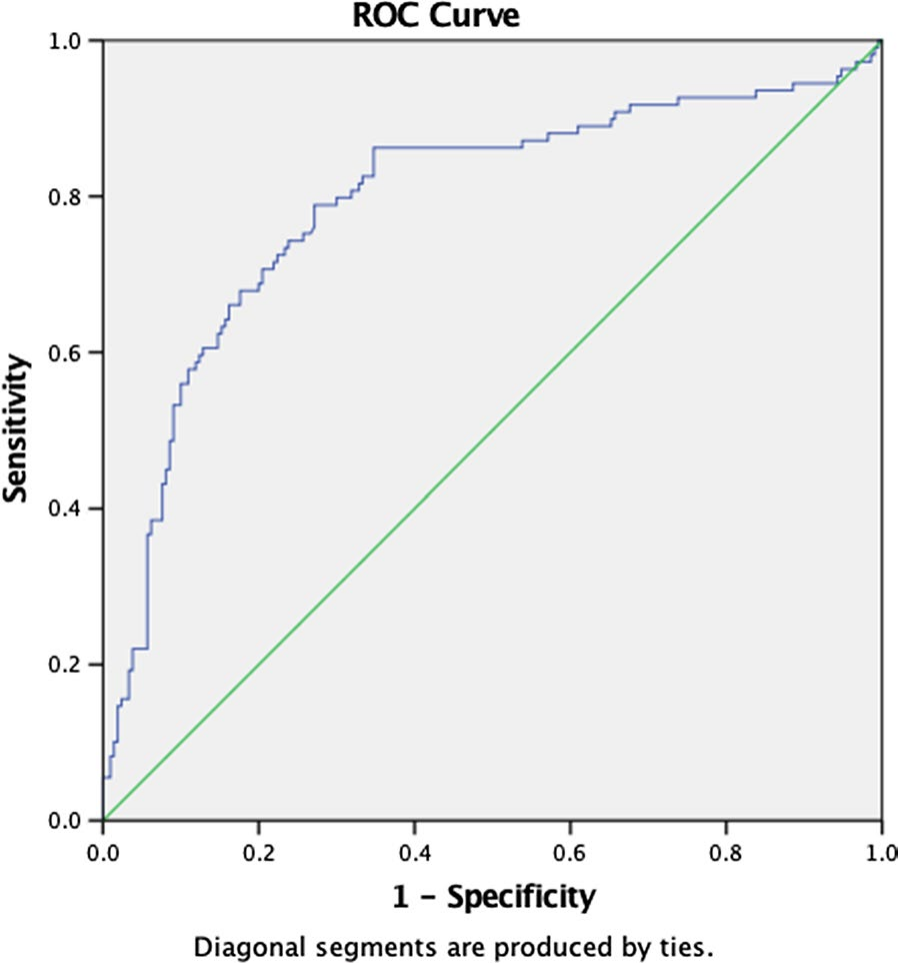
Receiver operating curve of red cell distribution width (RDW) and mean platelet volume (MPV) in patients diagnosed with active ocular manifestations of Behçet’s disease

The frequencies of different manifestations of active BD are listed in Table 2. One hundred sixty-one patients (50.4%) were found to have active BD manifestations in their most recent examination. Among 109 (31.4%) patients with active ocular involvement, anterior uveitis was observed in 94 (29.5%), and posterior uveitis in 17 (5.4%). Retinal vasculitis was seen in 58 (18.2%) patients. Active oral aphthae were present in 59 patients (18.5%), and genital aphthae were found in 9 (2.8%). Patients with active BD had significantly higher RDW (*p* < 0.001) and MPV (*p* < 0.001) in comparison to patients without any active manifestation (Fig. 1). Similarly, patients who had ocular manifestations in any site of involvement had higher RDW (*p* < 0.001) and MPV (*p* < 0.001) (Fig. 2). The presence of other BD manifestations, however, was not associated with MPV or RDW values in comparisons of patients with active BD vs. those whose disease was in an inactive state.

**Table 2:** Mean platelet volume (MPV) and red blood cell distribution width (RDW) values according to Behçet’s disease activity status

BD active manifestations		Frequency (%)	MPV (mean ± SD)	*p*	RDW (mean ± SD)	*p*
Active BD	No	119 (37.4%)	9.37 ± 1.37	< 0.001	13.26 ± 0.76	< 0.001
	Yes	200 (62.6%)	11.05 ± 2.45		14.38 ± 1.47	
Active ocular signs	No	210 (65.9%)	9.82 ± 1.84	< 0.001	13.56 ± 1.09	< 0.001
	Yes	109 (34.1%)	11.59 ± 2.53		14.72 ± 1.5	
Active oral signs	No	206 (64.5%)	10.38 ± 2.3	0.63	13.96 ± 1.36	0.63
	Yes	113 (35.5%)	10.51 ± 2.19		13.95 ± 1.36	
Active genital signs	No	310 (97.1%)	10.47 ± 2.26	0.87	13.96 ± 1.36	0.46
	Yes	9 (2.9%)	8.94 ± 1.59		13.89 ± 1.5	

BD, Behçet’s disease; MPV, mean platelet volume; RDW, red blood cell distribution width; SD, standard deviation

### Predictive value of RDW and MPV for BD manifestations

Separate binomial logistic regression analyses were used to determine whether RDW and MPV could indicate the likelihood of patients having active BD or having active ocular manifestations of the disease. The results produced a statistically significant logistic regression model in which the weight of RDW and MPV had some predictive power for active BD status, with χ^2^ (2) = 82.205 and *p* < 0.001. The model explained 31.0% (Nagelkerke R2) of the variance in BD, and correctly classified 68.3% of cases. Sensitivity was 80% and specificity was 51%, with a positive predictive value of 62% and a negative predictive value of 71%. Both of these two predictor variables were statistically significant (Table 3). Higher RDW and MPV were associated with a higher potential incidence of BD. The area under the ROC curve was 0.785 (95% confidence interval [CI] 0.737–0.834), which is considered an acceptable level of discrimination.

**Table 3:** Linear regression analysis of the correlation of mean platelet volume (MPV) and red blood cell distribution width (RDW) with Behçet’s disease and ocular manifestations

Condition	Variable	B	SE	Wald	df	*p*	Odds ratio	95% CI for odds ratio	
								Lower	Upper
Active BD	RDW	0.709	0.131	29.362	1	< 0.001	2.031	1.572	2.625
	MPV	0.32	0.07	21.221	1	< 0.001	1.378	1.202	1.579
	Constant	− 12.472	1.893	43.392	1	< 0.001	0		
Ocular BD (active)	RDW	0.584	0.112	27.382	1	< 0.001	1.793	1.441	2.232
	MPV	0.289	0.063	20.993	1	< 0.001	1.335	1.18	1.511
	Constant	− 11.965	1.626	54.148	1	< 0.001	0		

BD, Behçet’s disease; MPV, mean platelet volume; RDW, red blood cell distribution width; SD, standard deviation

The highest rates of sensitivity and specificity derived using Youden’s J were found for the cut-off points of diagnostic accuracy for RDW and MPV. The RDW value of 14.15 showed 77% sensitivity and 65% specificity for the diagnosis of active BD. The cut-off value of MPV was 12.95, with 73% sensitivity and 63% specificity.

The relationship between RDW and MPV and active ocular manifestations was evaluated with binomial logistic regression. The results yielded a statistically significant model, with χ^2^ (2) = 76.948 and *p* < 0.001. The model explained 29.6% (Nagelkerke R2) of the variance in active ocular manifestations of BD, and correctly classified 77.7% of patients. Sensitivity was 53%, specificity was 90%, positive predictive value was 74%, and negative predictive value was 79%. In the predictive model, RDW and MPV were statistically significant (Table 3), with higher values indicating the potential incidence of active ocular manifestations of BD. The area under the ROC curve was 0.794 (95% CI 0.737–0.850), which was considered an acceptable level of discrimination. According to analysis with Youden’s J, the cut-off values for a diagnosis of active ocular BD were 14.93 for RDW (62% sensitivity and 92% specificity), and 13.42 for MPV (52% sensitivity and 90% specificity).

### Relationship of RDW and MPV with IBDDAM score

Multiple regressions were run to predict overall IBDDAM score from the patients’ age, gender, body mass index (BMI), MPV, RDW, DMARD use, active BD status, active ocular signs, and active oral and genital ulcers. Six of the variables, e.g. age, gender, BMI, BD activity status, and active oral and genital aphthae, had no statistically significant role in the model. After omitting these variables from the predictive model, partial regression plots revealed linearity and yielded a plot of studentized residuals against the predicted values. The residuals were independent, according to the Durbin–Watson statistic of 1.925. The multiple regression model showed statistically significant predictive power for IBDDAM score, with F (4, 314) = 26.06, *p* < 0.001, and adjusted R^2^ = 0.24. All four variables added statistically significant value to the prediction. The regression coefficients and standard errors are reported in Table 4.

**Table 4:** Summary of multiple regression analysis

Variable	B	SE beta	Beta	*p*
Intercept	30.154	8.68		
RDW	2.07	0.653	0.166	0.002
MPV	0.888	0.394	0.118	0.025
Active ocular BD	4.143	1.75	0.116	0.019
DMARDs	13.793	1.704	0.4	0.001

BD, Behçet’s disease; DMARDs, disease-modifying anti-rheumatic drugs; MPV, mean platelet volume; RDW, red blood cell distribution width; SE, standard error

## Discussion

The present study aimed to investigate the correlation between two laboratory parameters which are easily obtainable from a blood sample and BD activity along with its different manifestations. RDW was significantly higher in patients with ocular and oral manifestations of BD, regardless of disease activity. MPV, however, was not associated with clinical manifestations of BD. The results also demonstrated that RDW and MPV correlated with the activity of BD, particularly in patients with ocular manifestations. According to Aksoy et al. study RDW were significantly higher in patients with Behçet's disease than in controls, and in those with active disease compared with inactive disease or controls. No differences were observed in RDW between patients with or without ocular or vascular involvement [19].

No such correlation was found between MPV or RDW and other BD signs and symptoms such as genital ulcers or vasculitis, probably because the incidence of these manifestations was low in our population sample. The area under the ROC curve indicated acceptable discriminative ability for RDW and MPV in differentiating active BD and active ocular manifestations of the disease. A linear correlation was also found between overall IBDDAM score and the aforementioned parameters.

Since its introduction, BD diagnosis has been based primarily on clinical assessment and physical examination, and no specific paraclinical findings or laboratory tests have been especially applicable in the process [20]. In response to the need for better diagnostic methods and in order to search for a link that connects paraclinical methods to disease activity, several biomarkers and factors have been identified as associated with BD activity, including platelet-to-lymphocyte ratio (PLR), neutrophil-to-lymphocyte ratio (NLR), and erythrocyte sedimentation rate (ESR) [21, 22]. Earlier studies have detected increased MPV and RDW in patients with inflammatory and chronic diseases, and researchers have speculated that these biomarkers might be associated with inflammation and pro-inflammatory processes [23–25]. In this connection, RDW was found to correlate with levels of inflammation in rheumatoid arthritis, inflammatory bowel disease, and ankylosing spondylitis [16, 17, 24, 26]. Furthermore, MPV has also been suggested as a biomarker associated with thrombosis and oral ulcers in patients with BD [27].

Since only a few studies have investigated the importance of these easily-applicable and inexpensive biomarkers in the clinical assessment of patients with BD, it has not been possible to use these biomarkers in clinical approaches. Nonetheless, our results show elevated RDW and MPV not only during BD flares but also in association with its ocular manifestations. Although the underlying etiology for this phenomenon is not well understood, we speculate that with the progression of pro-inflammatory states and the resulting disruption of erythropoiesis, secretion of inflammatory cytokines by platelets is enhanced, and an increase in RDW and MPV follows [12, 19]. Medication with DMARDs might also contribute to myelosuppression and result in disrupted CBC parameters such as RDW and MPV [28].

Several recent studies have noted a significant increase in RDW in patients with a history of BD regardless of disease activity [19, 29, 30]. Uzkeser et al. found increased MPV in patients with BD compared to a healthy control group, along with a significant positive correlation between MPV and BD manifestations such as oral and genital ulcers, ocular involvement, and arthritis [31]. In contrast, we found no significant association between active oral and genital aphthae and MPV; however, we did find that MPV correlated with ocular involvement in BD. These contrasting results might be due to the low incidence of involvement of other organs in BD in the present study population.

In 2013, Turkcu et al. claimed that MPV measurement had no predictive value in determining the likelihood clinical improvement in ocular BD after standard immunosuppressive treatment [32]. Although we found no significant difference in MPV values between patients with and without ocular BD, the results of our study show not only a correlation between MPV and DMARD use, but also increased MPV in patients with active ocular involvement.

To our knowledge, our study is one of the first to evaluate RDW and MPV changes in patients with BD in connection with their disease activity and current manifestations. Additionally, we assessed the possible relationships of BD flares with demographic and laboratory characteristics, although in line with previous studies, no significant correlations could be found [9].

Aksoy et al. reported in 2015 that MPV was not elevated in patients with BD compared to healthy individuals, or in patients with active BD compared to patients with inactive BD, despite finding higher MPV values in patients with vascular involvement than those without [19].

It is well established that platelets play an essential role in various inflammatory diseases by stimulating immune responses and activating immune cells, which is in line with our finding of significantly higher MPV values in patient with BD, among whom the increase was higher in patients with active BD.

The present findings point to MPV and RDW as potentially promising clinical tools in the diagnosis of BD, particularly in its active period. In addition, MPV and RDW might be applicable as screening parameters in patients with BD, although this requires further comprehensive research with prospective studies and randomized trials.

In light of the present results and previous finding regarding specific associations of NLR and PLR with the active phase of BD, we recommend further investigation into the correlation of PLR and NLR ratios with different clinical manifestations of Behçet’s disease.

## Limitations

The present study was not without limitations. Because it was carried out at a single center and conducted with a cross-sectional study design rather than a cohort design, our results provide some evidence for the applicability of MPV and RDW as a variables able to predict the disease course and its manifestations, yet further studies are required to test these parameters and establish them as valuable tools for the diagnosis and monitoring of patients with BD. Another limitation of our study was that laboratory tests were done only once for each patient regardless of their clinical status or disease activity. In addition, the absence of data on dietary habits limited our results. Finally, because of the low incidence of some signs and symptoms of BD in our study population, we were not able to evaluate the involvement of all organs with the same level of reliability.

## Conclusion

BD was found to be associated with an increase in MPV and RDW, particularly during active phases. RDW and MPV were also found to have predictive value for screening to detect BD activity and its ocular complications.

## Data Availability

The datasets used and analyzed in the current study are available from the corresponding author on reasonable request.
